# SG-APSIC1111: Abstract management services for the sterilization of single-use medical supplies

**DOI:** 10.1017/ash.2023.102

**Published:** 2023-03-16

**Authors:** Tussanee Nimnaparoj

**Affiliations:** Central Sterilizing Services Association, Bankok, Thailand

## Abstract

**Objectives:** Single-use medical supplies are usually expensive, resulting in excess costs for hospitals and patients. Reusing single-use medical devices by resterilization or reprocessing has thus been enacted. We compared the cost of resterilizing single-use medical supplies with the cost of new purchases to reduce the unnecessary resterilization of medical supplies. **Methods:** The central sterile supply department (CSSD) listed single-use medical supplies that were sent for resterilization. Policies and guidelines for reusing or resterilizing single-use medical supplies were established following the standards for disinfection and sterilization. The costs of the resterilizing process for single-use medical supplies were compared with the expenses of new purchases of those medical supplies. **Results:** In 2019, many medical supplies were resterilized, and the resterilization of single-use devices cost up to 2,352,270 Thai baht (US $68,340). Since this project was implemented in fiscal year 2020 (October 1, 2019–September 30, 2020), the resterilization of medical supplies has decreased, and the cost of resterilization has decreased to 1,356,280 Thai baht (US $40,557), leading to a saving of 995,990 Thai baht (US $29,783, or 42% of the resterilization cost in 2019). The CSSD proposed a resterilization policy in which resterilization for reuse must be done for medical supplies and/or devices that cost ≥1,000 Thai baht (US $30). **Conclusions:** Although this project did not reach the target outcome of 100% reduction, the outcome was consistent with the aims of the project. The cost of resterilization of single-use medical supplies can be reduced, and a resterilization system can be developed that assures safety and effectiveness to both service providers and patients.

